# Towards social autonomous vehicles: Efficient collision avoidance scheme using Richardson’s arms race model

**DOI:** 10.1371/journal.pone.0186103

**Published:** 2017-10-17

**Authors:** Faisal Riaz, Muaz A. Niazi

**Affiliations:** 1 Dept. Of Computing-Iqra University, Islamabad, Pakistan; 2 Dept. Of Computer Sciences-COMSATS, Islamabad, Pakistan; Chongqing University, CHINA

## Abstract

This paper presents the concept of a social autonomous agent to conceptualize such Autonomous Vehicles (AVs), which interacts with other AVs using social manners similar to human behavior. The presented AVs also have the capability of predicting intentions, i.e. mentalizing and copying the actions of each other, i.e. mirroring. Exploratory Agent Based Modeling (EABM) level of the Cognitive Agent Based Computing (CABC) framework has been utilized to design the proposed social agent. Furthermore, to emulate the functionality of mentalizing and mirroring modules of proposed social agent, a tailored mathematical model of the Richardson’s arms race model has also been presented. The performance of the proposed social agent has been validated at two levels–firstly it has been simulated using NetLogo, a standard agent-based modeling tool and also, at a practical level using a prototype AV. The simulation results have confirmed that the proposed social agent-based collision avoidance strategy is 78.52% more efficient than Random walk based collision avoidance strategy in congested flock-like topologies. Whereas practical results have confirmed that the proposed scheme can avoid rear end and lateral collisions with the efficiency of 99.876% as compared with the IEEE 802.11n-based existing state of the art mirroring neuron-based collision avoidance scheme.

## Introduction

Road collisions are an inevitable element of human life. Riaz and Niazi [1] have presented literature showing that road collisions have the potential of becoming the 5th major cause of human deaths by the 2030. According to Gopinath et al. [2], road injuries are considered to be a twelfth main reason of human disability. From these facts, it can be implied that road collisions cannot be avoided, but they can certainly be limited with the help of latest advances in the field of Intelligent Transport System (ITS) such as by means of methods in the domain of Autonomous Vehicles (AVs).

Autonomous vehicles can help in avoiding the road collisions. According to Macy et al. [3], AVs do not drink or distract like human drivers and have fewer chances of accidents as compared to the human-driven vehicles. Techniques for improving road problems can ranging from static such as using ant-based methods [4] to real-time such as AVs. Furthermore, Baskar et al. [5] noted that the number of collisions can be decreased by introducing inter-AVs and Road Side Units (RSUs) based communication capabilities. However, the most of the research in the context of collision avoidance has been performed to address, separately, the rear end, front end and lateral collisions in less congested and with high inter-vehicular distances. Whereas the flock like topology, a typical scenario of urban traffic single one-way lane, where the traffic pattern is congested, the inter-vehicular distance is small, and the chances of the rear end, front end and lateral collisions is very high has not been addressed sufficiently. While literature has shown that agents can can self-enforce agreements [6], such agent-based techniques have not previous been exploited in this domain. Furthermore, the collision avoidance capabilities of AVs have been improved by using different methodologies, however, human-inspired designs have not been explored in this context, especially the human brain parts that are involved in the human-human interaction, which make them social and help them in understanding and adapting the behaviour of other humans.

Humans are inherently social because of the way the human brain is structured [7]. According to Tramacere and Ferrari [8], humans use mentalizing and mirroring functions, imparted in their brains, to recognise and adapt the behaviours of other humans and hence make them social. The purpose of the mentalizing part is to recognise the intention of other humans [9], whereas the MNS is responsible for helping a person to copy the actions of another person [10]. It would be interesting to evaluate the mentalizing and mirroring concepts after incorporating them in the AVs to enhance their collision detection and avoidance capabilities.

Now the question arises that what is the benefit of making AVs social. According to Bicchi and Tamburrini [11], agents are social when they share the same space. In our case when the AVs will travel in a flock like topology by sharing the highly congested urban road, then they can be perceived as social agents and hence need some mechanisms that help them to avoid the collisions using human inspired social mechanism. However, to authors’ best knowledge, AVs have not been designed yet as social agents. According to Libero et al. [12], the ability to interpret agents’ intent of their actions is a vital skill in a successful social interaction and can be explored to enhance the pre-crash sensing and avoidance capabilities of AVs by making quick decisions in short reaction time. However, this line of research has not been also explored in the case of AVs that help them to be social and understanding the dangerous intents of other AVs and furthermore to avoid collisions. To address this issue, We have designed our social agent having the capabilities of mentalizing and mirroring and for this purpose we utilized Exploratory Agent Based Modeling (EABM) level of Cognitive Agent Based Computing (CABC) framework proposed by Niazi and Hussain [13].

*Contribution*: In this paper, following contributions have been made.

- A Novel architecture of social agent inspired by modified Richardson’s Arms Race model has been proposed to enhance the collision avoidance capabilities of AVs in flocks like topologies.
- Practical implementation of Social AV and the real time validation of the collision avoidance capabilities of proposed social agent in a flock like topology.

The rest of the paper is organised as follows. Section 2 presents the literature review. Section 3 presents the proposed social agent architecture along the Richardson’s Arm race-based mathematical modelling of its social components. A simulation environment, simulation/real field experiments and its results and discussion have been presented in section 4. Furthermore, the comparison with existing state of art has also been made in section 4. Section 5 concludes the paper.

## Related work

Existing literature justify the need of automated vehicles according to different aspects. In this modern world, fuel consumption is very high, which causes the depletion of natural resources like petrol, diesel and gasoline. According to Li et al. [14], the autonomous vehicles can be used to decrease the fuel consumption by utilizing stabilizing periodic control method within the autonomous vehicles travelling in a platoon formation. Furthermore, Hu et al. [15] have also justified the need of autonomous vehicles for efficient fuel consumption and high road safety. For this purpose, the authors have proposed a model predictive fuel-optimal controller, which helps in optimizing the vehicle speed and ultimately less fuel consumption. According to Lu et al. [16], the AVs equipped with Electronic Stability Control (ESC) have proven their worth as compared to the conventional cars in high road safety.

**Fig 1:**
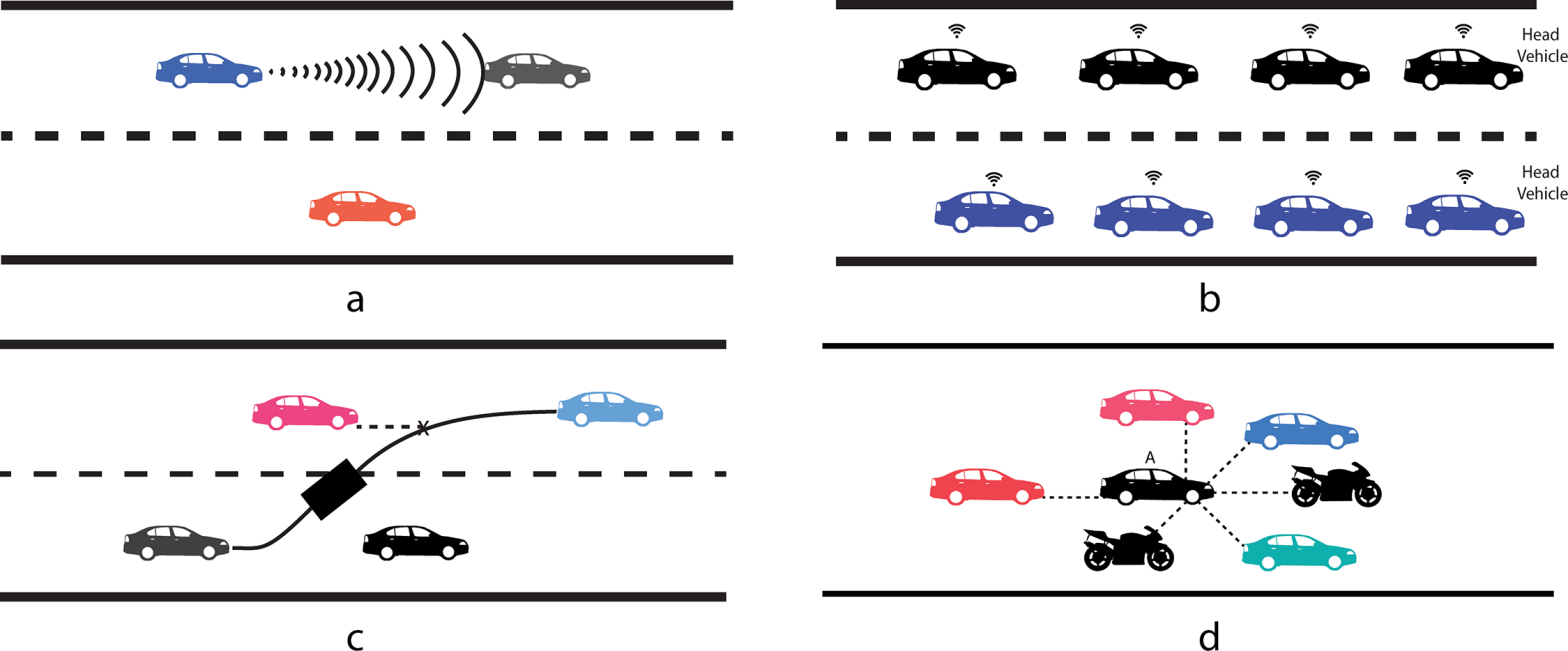
Collision avoidance scenarios addressed extensively in literature. (a) Rear end collision avoidance, (b) Cooperative collision avoidance in Adaptive Cruise Control or Platooning, (c) Lane departure or Lateral collision avoidance, (d) Flock like topology (A typical scenario in congested urban road).

According to the literature, the research on collision avoidance has been performed to address the three types of scenarios presented in Fig 1A, 1B and 1C. The scenario presented in Fig 1A is presenting rear end collision avoidance using onboard sensors such as sonars, Light Detection and Ranging (LIDAR), and cameras, whereas the scenario presented in Fig 1B, is depicting the collision avoidance in the context of platooning and Adaptive Cruise Control (ACC) using cooperative communication approach. To address the scenario presented in Fig 1A, many rear end collision avoidance solutions based on onboard sensors or wireless communication have been proposed. Gracia et al. [17], proposed sliding mode control based rear end collision avoidance solution. Sato and Akamatsu [18], modelled the human driver characteristics like driving style, reaction time and cognitive state using fuzzy logic to propose the rear end collision avoidance scheme. Li et al. [19], proposed GPS enabled rear end crash warning system using DSRC based inexpensive high-end devices. In the literature, researchers have addressed platooning and ACC, Fig 1B, scenarios with extensive research work. In this regard, Liu and El Kamel [20] have proposed a decentralised cooperative adaptive cruise control algorithm using V2X communication. Milanés et al. [21] have proposed Cooperative Adaptive Cruise Control (CACC) in Real Traffic Situations using Vehicle-2-Vehicle (V2V) communication. The third main scenario, which has been addressed by various researchers, is lane departure/ lateral collision avoidance as shown in Fig 1D. In Schwindt et al. [22], a lane departure warning system is proposed using left, right, rear and forward sensors, a direction sensor, a processing unit, memory, and I\O interface. In other research work, the cognitive automatic overtaking system using vision system and fuzzy logic based controller is proposed to avoid the lateral collisions during overtaking manoeuvres by Milanes et al. [23]. However, the scenario depicted in the Fig 1D has been ignored at large. The presented scenario depicts the vehicle travelling at low speed in congested urban traffic on the single one-way road. If we see the details of the scenario, then it looks like a flock of vehicles, which are travelling in the same direction on a congested road and their ultimate goal is to reach their destinations safely.

If we consider the vehicle A, Fig 1D, as an autonomous vehicle, then it needs a robust motion controller, which helps AV to travel safely by avoiding front end, rear end and lateral collisions. The need of robustness is due to heavily congested traffic, which decreased the inter-vehicular distance to dangerous limits. So the awareness of neighbouring vehicles’ position and quick reaction time is the key to avoid the collisions efficiently. During the literature review, we tried our best to find such a published work that addresses this issue with sufficient details and practical validation approach, but to our best knowledge, no such work has been reported. Then we analysed the above-mentioned research ideas, which have been done to address the scenarios 1a, 1b, and 1c but we found them unsuitable to address the scenario depicted in Fig 1D. The rear end collision avoidance solutions provided to address the scenario 1a has the following issues in this context. The mathematical based solutions provided by [17] is highly dependent on precise mathematical models as noted by [24] and has not been modelled by keeping in view the nonlinear factors like road traffic pattern and driver reaction time. Whereas, the fuzzy logic based solution, provided by [18] rely on the number of fuzzy rules and an excessive number of such will straightforwardly prejudice its efficiency in terms of delayed reaction time. The solutions provided for scenario 1b are using DSRC based V2V or V2I communication, whereas DSRC has been proved to be failed due to long packet delay and communication failure in congested urban traffic. In the same way, the solutions provided for scenario 1c are using fuzzy logic or wireless communication that are not suitable to address the problems associated with scenario 1d. Furthermore, all of the above-mentioned solutions address rear end, front end and lateral collisions separately. No such framework is available that helps the AV to avoid the rear end as well as lateral collisions at the same time with the quick reaction in the scenario of heavily congested urban traffic. This research gap motivates us to propose a novel scheme that helps the AV to travel in a flock like topology, which is very common in 3rd world countries such as Pakistan, India, Bangladesh and Srilanka. To cover this research gap, we explored the concept of social AVs. In existing literature following efforts have been made in this context. Bicchi and Tamburrini [11] devised the collision avoidance mechanism in the artificial society of robots by making them social. Each robot keeps track of its neighbouring robots, same as humans follow social rules and avoid collisions in crowded spaces, and adapt collision avoidance strategy accordingly. Furthermore, the authors have suggested that such teams of robots can be built by following human social life protocols that help them to coexist and move safely. According to [25] in near future, AVs will share the road with other road commuters and will become the part of a complex social-technical system. To be socially accepted in this complex sociotechnical system, AVs need novel AV➔X, X = {Human driven vehicles, pedestrians, other AVs}, interaction protocols. Furthermore, the authors have declared AVs as embodied intelligent agents. However, in this research work authors have just presented the theoretical concept of making AVs social and no practical steps have been taken in this regard. According to [26], a new generation of robots has a need for the social mechanisms that help them to engage the post-stroke patients in a better way. It has been noted by the authors that creating robots that have the capability to adapt their behaviour according to the personality of patients is a difficult task. In this regard, they have proposed a learning algorithm using policy gradient reinforcement learning (PGRL). Li et al. [27] have evaluated the role of social robots as an online instructor, which teach the students through videos. The concept of social learning using different machine learning algorithms has been discussed by [28] to make the autonomous vehicles a valuable part of the society. Furthermore, the author has argued that autonomous vehicles will become more resilient by adopting the paradigm of social learning. Another interesting research work, which supports our argument of using social aspects in autonomous cars is presented by [29]. According to the authors, AVs can be made social by connecting the nodes with each other, which will help the AVs to enhance their trust on each other by the behaviour they exhibit with each other. A concept of socially behaving autonomous vehicles has been introduced by [30].

## Proposed social agent architecture using EABM modeling

**Fig 2:**
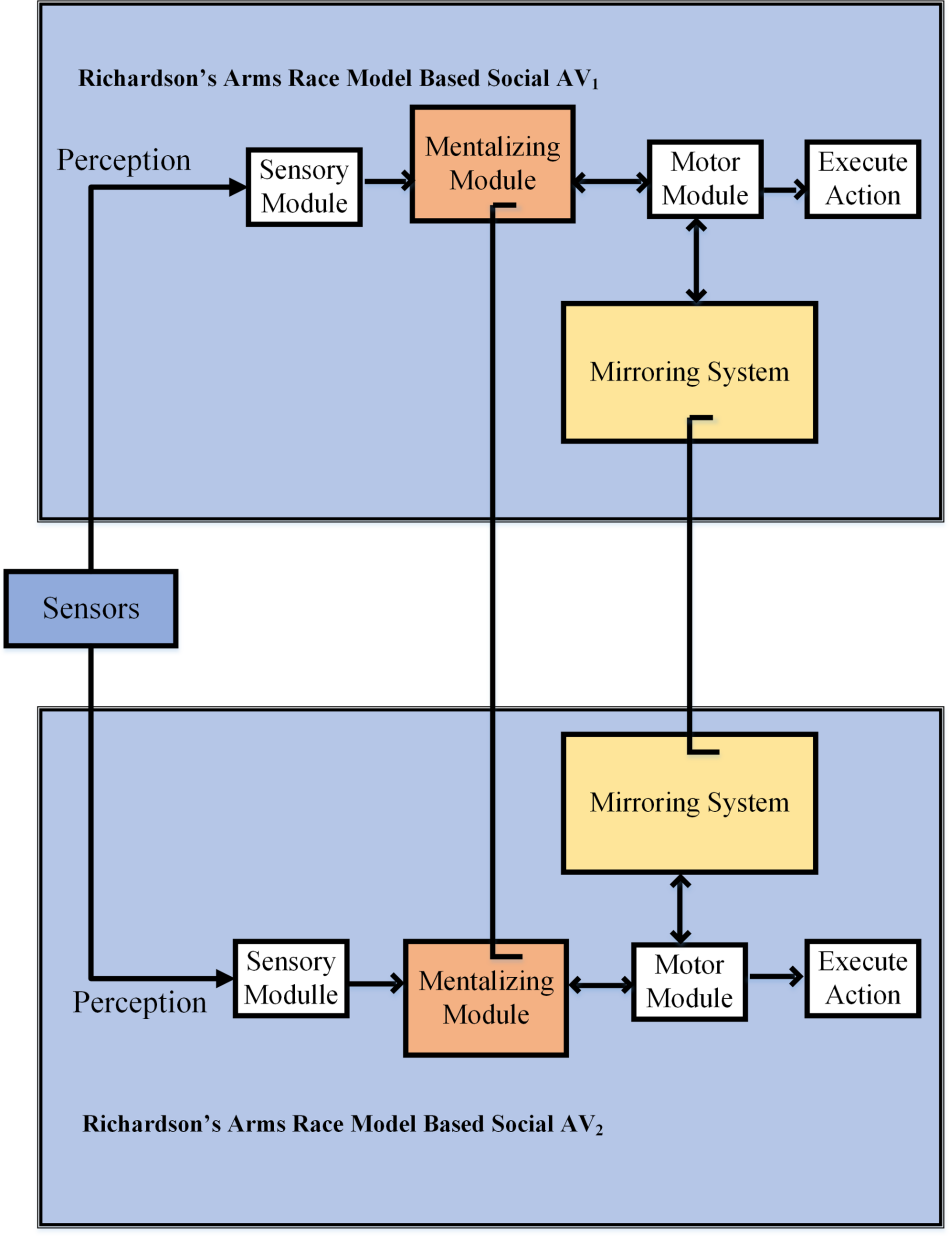
AVs installed with proposed social agents interacting socially with each other.

As mentioned earlier in introduction section that our AV is designed inspired by the human capability of monitoring their neighbours and then adapting the same moves as their neighbours. We have utilized exploratory agent based modelling level of the CABC framework to explore the human brain inspired mechanism in the design of our social agent. The proposed agent is envisaged to avoid road accidents by keeping track of their neighbouring AVs and then performing the same manoeuvre as they do. The proposed agent possesses the ability to react in the event of danger inspired by human brain capable of mirroring and is proposed to be housed inside the vehicle. Recall that the agent is responsible for detecting potential threats and take necessary actions if required. The architecture of the proposed agent is presented in Fig 2.

### Description of the agent

It can be seen from the Fig 2 that the proposed architecture consists of five main modules.

- **Sensory Module**: It keeps track of the distance between neighbouring cars on a road segment.
- **Mentalizing Module:** This module helps the AV to find the intention of neighbouring AVs. To find out the intentions, Richardson’s arms race model Eqs 1 and 2, presented in section 6, have been employed. The mentalizing module keeps sensors data to find out the current motion pattern, which helps the AV to predict the potential collision threat in advance.
- **Mirroring Module:** The mirroring module helps the AV to change its trajectory according to the changed trajectory of the nearest AV. To create the capability of mirroring in AVs, we have utilized the Eqs 3 to 7 of the modified Richardson’s arms race model.
- **Motor Module**: This module will initiate the execution module to execute the mirroring instructions, adapted angle and speed.
- **Execute Action Module**: This module will act in the place of the human driver to perform accident avoidance manoeuvre.
- The Fig 2, is presenting the interaction between two AVs using proposed agent architecture. Both AVs keep track of each other’s movement intentions and avoid collisions using mirroring option.

### Proposed Richardson’s arm race-based mathematical modelling

It has been noted earlier in the previous sections that the proposed social agent incorporates the notion of intention understanding and adapting the behaviour of neighbouring AVs. In order to express, these capabilities of social agent mathematically, the Richardson’s arms race model is employed [31]. The Richardson’s model, which studies the circumstances under which two nations can avoid war, uses a set of linear differential equations. This work uses the said model to formulate the generation of fear in the proposed social agent. As shown in Fig 2 both social agents of the two vehicles are exchanging their positions to evade the chance of an accident using distance-measuring sensors.

Let us consider a set of vehicles ***V = {v***_***i***_***}*, *where i = 1*, *2*, *3*…*n*** is the set of vehicles installed with social agents belong to the set of ***Social_Agent = {SA***_***j***_***}*, *where j = 1*, *2*, *3…n*** are travelling on an urban road. Let us further consider a case when two vehicles v_1_ and v_2_ ∈ V are moving side by side on the road very close to each other. Whereas, the position of v_1_ at time n_1_ with respect to v_2_ is represented by v_1_ (n). Similarly, v_2_ (n) represents the position of v_2_ at the time n_1_ with respect to v_1_.

It has been further supposed that the social agent is equipped with a buffer, which maintains the different changes in the lateral position of v_1_ and v_2_: Like

$$\begin{array}{l} \;\Delta v_{1}\left(n_{1}\right)=v_{1}\left(n_{1}\right)-v_{1}\left(n_{1}-1\right)\;\left\{Change\;in\;the\;lateral\;position\;of\;v_{1}w.r.t\;v_{2}\;at\;time\;n_{1}\right\}\\ \Delta v_{1}\left(n_{2}\right)=v_{1}\left(n_{2}\right)-v_{1}\left(n_{2}-1\right)\;\left\{Change\;in\;the\;lateral\;position\;of\;v_{1}w.r.t\;v_{2}\;at\;time\;n_{2}\right\}\\ \;\vdots \;\vdots \;\vdots \\ \;\Delta v_{1}\left(n_{i}\right)=v_{1}\left(n_{i}\right)-v_{1}\left(n_{i}-1\right)\;\left\{Change\;in\;the\;lateral\;position\;of\;v_{1}w.r.t\;v_{2}\;at\;time\;n_{i}\right\} \end{array}$$

The SA_1_ ∈ Social_Agent compare the values of these changes in position variables. If the value of Δv_1_(n_1_) < Δv_1_(n_2_) and Δv_1_(n_2_) < Δv_1_(n_3_) is true, then it means v_2_ is approaching to the safety distance and in this way social agent predict the malicious intent of neighbouring vehicle. Hence the mentalizing procedure for v_1_ and v_2_ can be defined by Eqs 1 and 2 respectively.

$$\Delta v_{1}(n_{i})=v_{1}(n_{i})-v_{1}(n_{i}-1)$$ (1)

$$\Delta v_{2}(n_{j})=v_{2}(n_{j})-v_{2}(n_{j}-1)$$ (2) (Mentalizing)

The Eqs 1 and 2 help the proposed agent installed on v_1_ and v_2_ in performing their mentalizing function, respectively, i.e. it helps in assessing the future intention and, motion trajectory of the nearest neighbouring vehicle.

After assessing the relative position of nearest neighbours, there will be a need to execute the safety manoeuvres. However, the question arises what should be the nature of safety manoeuvres. Here Eq 3 comes, which helps the proposed social agent in performing its mirroring function. Hence,

$$\Delta v_{1}(n)=\delta_{1}\Delta v_{2}(\mathrm{n-}1)$$ (3) (Mirroring)

Where δ_1_ is referred as the position coefficient.

Note that the change in the position of v1 is limited by the road width.

$$\Delta v_{1}(n)=\delta_{1}v_{2}(n-1)-{\alpha_{1}v}_{1}(n-1)$$ (4)

$$\Delta v_{2}(n)=\delta_{2}v_{1}(n-1)-{\alpha_{2}v}_{2}(n-1)$$ (5)

Where α_1_and α_2_ are positive constants, representing the road capacity limits in terms of performing safety manoeuvres. Note that the intensity of fear experienced by a vehicle also depends on its type and size and is represented by g in Eqs 6 & 7. A lighter vehicle will have the higher fear intensity and vice versa.

The goal of the vehicle, which is ultimately its safety, has been represented by h. Now the Eqs 4 & 5 can be written as:

$\Delta v_{1}(n)=\delta_{1}v_{2}(n-1)-{\alpha_{1}v}_{1}(n-1)+g_{1}\;*h_{1}$ (6) (Mirroring final equation)

$$\Delta v_{2}(n)=\delta_{2}v_{1}(n-1)-{\alpha_{2}v}_{2}(n-1)+g_{2}*h_{2}$$ (7)

As we have seen, in Richardson’s construction of the model the parameters δ1, α1, g and h have very special meanings, which suggested that these constants should be positive. However, it has since been argued that negative parameters can have equally relevant interpretations and that both mathematically and substantively it makes more sense to consider a general model in which parameters are not constrained. We, therefore, rewrite (6) and (7) in a more standard form:

$$\Delta v_{1}(n)=\alpha_{1}v_{1}(n-1)+{\delta_{1}v}_{2}(n-1)+g_{1}*h_{1}$$ (8)

$$\Delta v_{2}(n)=\alpha_{2}v_{2}(n-1)+{\delta_{2}v}_{1}(n-1)+g_{2}*h_{2}$$ (9)

In addition, using Eqs 1 and 2, it can be written as

$$v_{1}(n)=(1+\alpha_{1})v_{1}(n-1)+{\delta_{1}v}_{2}(n-1)+g_{1}*h_{1}$$ (10)

$$v_{2}(n)={\delta_{2}v}_{1}(n-1)+(1+\alpha_{2})v_{2}(n-1)+g_{2}*h_{2}$$ (11)

If we define

$$(1+\alpha_{1})\equiv \beta_{1},(1+\alpha_{2})\equiv \beta_{2}$$

So

$$v_{1}(n)={\beta_{1}v}_{1}(n-1)+\delta_{1}v_{2}(n-1)+g_{1}*h_{1}$$ (12)

$$v_{2}(n)=\delta_{2}v_{1}(n-1)+{\beta_{2}v}_{2}(n-1){+g}_{1}*h_{1}$$

We have shown the formulation of the model for two vehicles only. The model can be extended for N vehicles in the future.

## Simulation/ Real time experiments along the result/ discussion and comparison with the existing state of the art

In this section, first of all, the simulation environment has been discussed. Afterwards, the simulation parameters and experimental design have been presented. Then the results of the simulation and real time experiments have been discussed. In the last, the comparison with the existing state of the arts has been performed.

### Simulation environment

**Fig 3:**
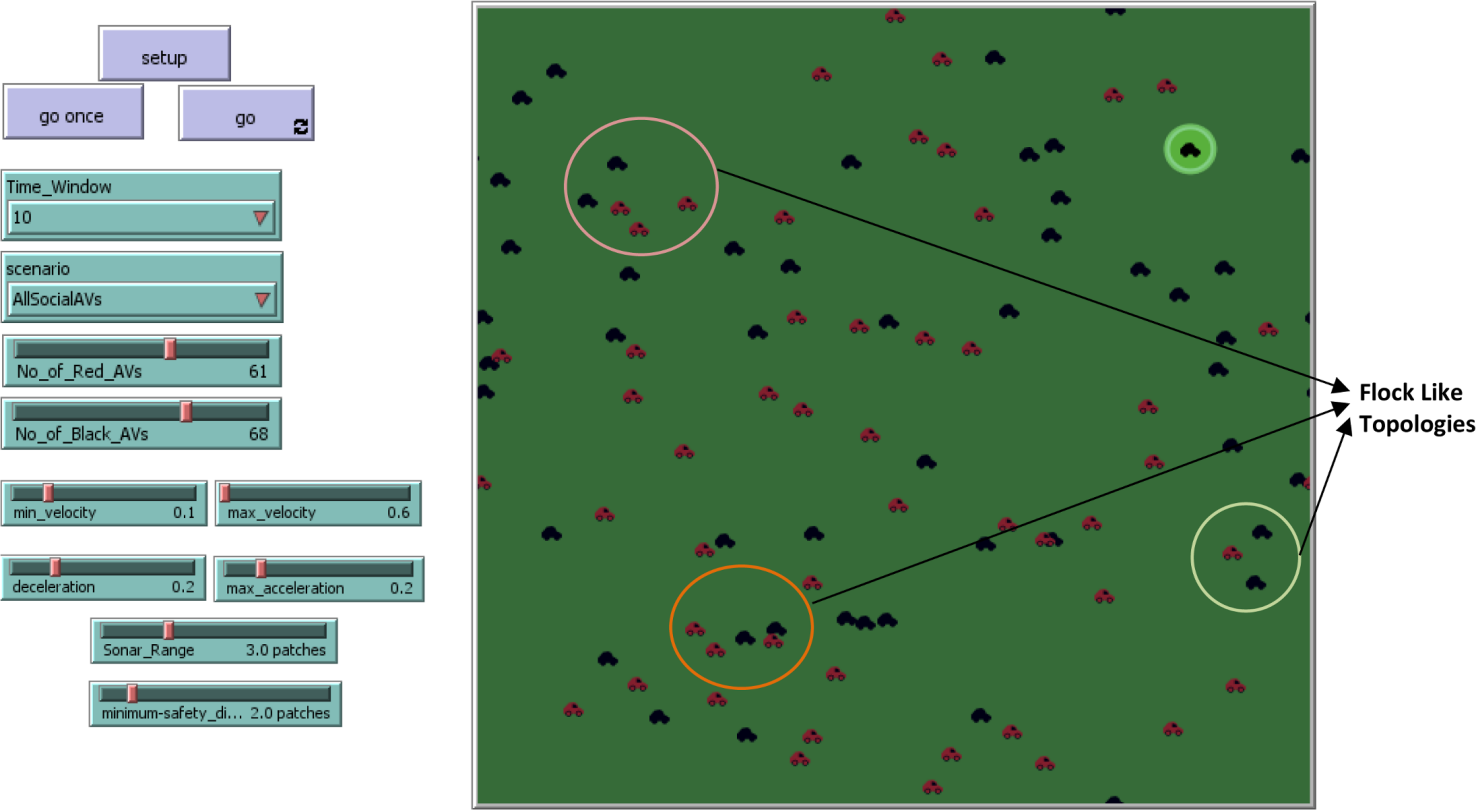
Main simulation screen of Richardson’s arms race model inspired agent-based collision detection and avoidance scheme.

The purpose of the research work is to introduce a social agent within the AV, having the capability of finding out the intentions of neighbouring AVs and avoiding the collisions. To simulate the concept of this agent-based system a standard agent-based simulation platform is the main requirement. For this purpose, Net logo 5.3 has been utilised which is a standard agent-based simulation environment. The Net Logo environment consists of patches, links, and turtles. Fig 3 presents the experimental environment along with input and output parameters. The left side of the simulation world contains input sliders and the right side is presenting the simulation world, executing the scenarios of AVs moving in a flock like topologies. It is important to mention here that the social agent installed in AVs have been designed with the help of Richardson’s arms race model, which were basically proposed to avoid the wars between two nations. Hence the red and black colors of AVs are depicting two different types of nations according to the description of Richardson’s arms race model.

#### Simulation parameters and experimental design

In this section, simulation parameters, Table 1, along with experimental design, Table 2, have been presented.

**Table 1:** Simulation parameters and their designated ranges.

Simulation Parameters	Range
(NO of Red AVs) & (NO of Black AVs	0–100
Sonar Range	0 to 10 meters
(Min Velocity) & (Max Velocity)	0 to 0.5 m/s & 0.6 to 1 m/s
(Acceleration) & (Deceleration)	0–1 m/s2 & 0–0.5 m/s2
Minimum Safety Distance	1.5 to 5 meters

**Table 2:** Test cases along the parameters and their corresponding values.

Exp #	Number of Red_AVs	Number of Black_AVs	Min Velocity Range	Max Velocity Range	Min Acceleration Rate	Deceleration Rate	Simulation Mode
1	40	40	0.3	0.3	0.1	0.1	Random Walk/ Social Agent
2	60	60	0.3	0.3	0.1	0.1	Random Walk/ Social Agent
3	80	80	0.3	0.3	0.1	0.1	Random Walk/ Social Agent
4	40	40	0.5	0.9	0.1	0.3	Random Walk/ Social Agent
5	60	60	0.5	0.9	0.1	0.3	Random Walk/ Social Agent
6	80	80	0.5	0.9	0.1	0.3	Random Walk/ Social Agent

In Table 2 the detailed experimental design has been proposed to test the performance of random walk based and Richardson’s arms race model installed AVs in terms of collision avoidance. The experimental set consists of 6 tests with same simulation parameters, but a different number of red and black AVs. The first three tests of experimental set help in testing the behaviour of AVs, in terms of collision avoidance for both random walk and Richardson’s arms race model, with low speed, minimum acceleration rate, minimum deceleration rate, minimum safety distance and low sonar range. Whereas the last three tests of experimental set help in testing the behaviour of AVs, in terms of collision avoidance for both random walk and Richardson’s arms race model, with high speed, minimum acceleration rate, minimum deceleration rate, minimum safety distance and low sonar range.

Further experimental set, presented in Table 3, has been proposed to find out the optimal speed, sonar range and safety distance, which can be adopted by the AVs to have the least collisions during their travel in the congested flock like topologies.

**Table 3:** Test cases to find optimal safety distances and sonar ranges.

Exp #	Sonar Range	Safety Distance	Min Velocity Range	Max Velocity Range
1	1	1	0.1	0.4
2	1	1	0.3	0.5
3	1	1	0.3	0.7
4	2	2	0.1	0.4
5	2	2	0.3	0.5
6	2	2	0.3	0.7
7	3	2	0.1	0.4
8	3	2	0.3	0.5
9	3	2	0.3	0.7

### Real time validation experimental design

To give the proof of concept and to perform the rigorous validation of the proposed social agent, we have performed field tests. For this purpose, a prototype AV platform has been built, which is equipped with sonar sensors and Arduino microcontroller. Furthermore, the functionality of the proposed social agent has been coded using the Integrated Development Environment of Arduino Microcontroller (IDEAM). The steps of real time experimental design are given as under.

(i) Three human-driven motorcycles manoeuvring around the prototype AV platform. The leading motorcyclist drives with different acceleration and deceleration rate. Whereas, the motorcyclists driving on both lateral sides drive with the same speed of AV and increase and decrease their lateral distance from AV in a random fashion.
(ii) The results of each test have been traced into a log file every millisecond.

### Results and discussion of simulation experiments

**Fig 4:**
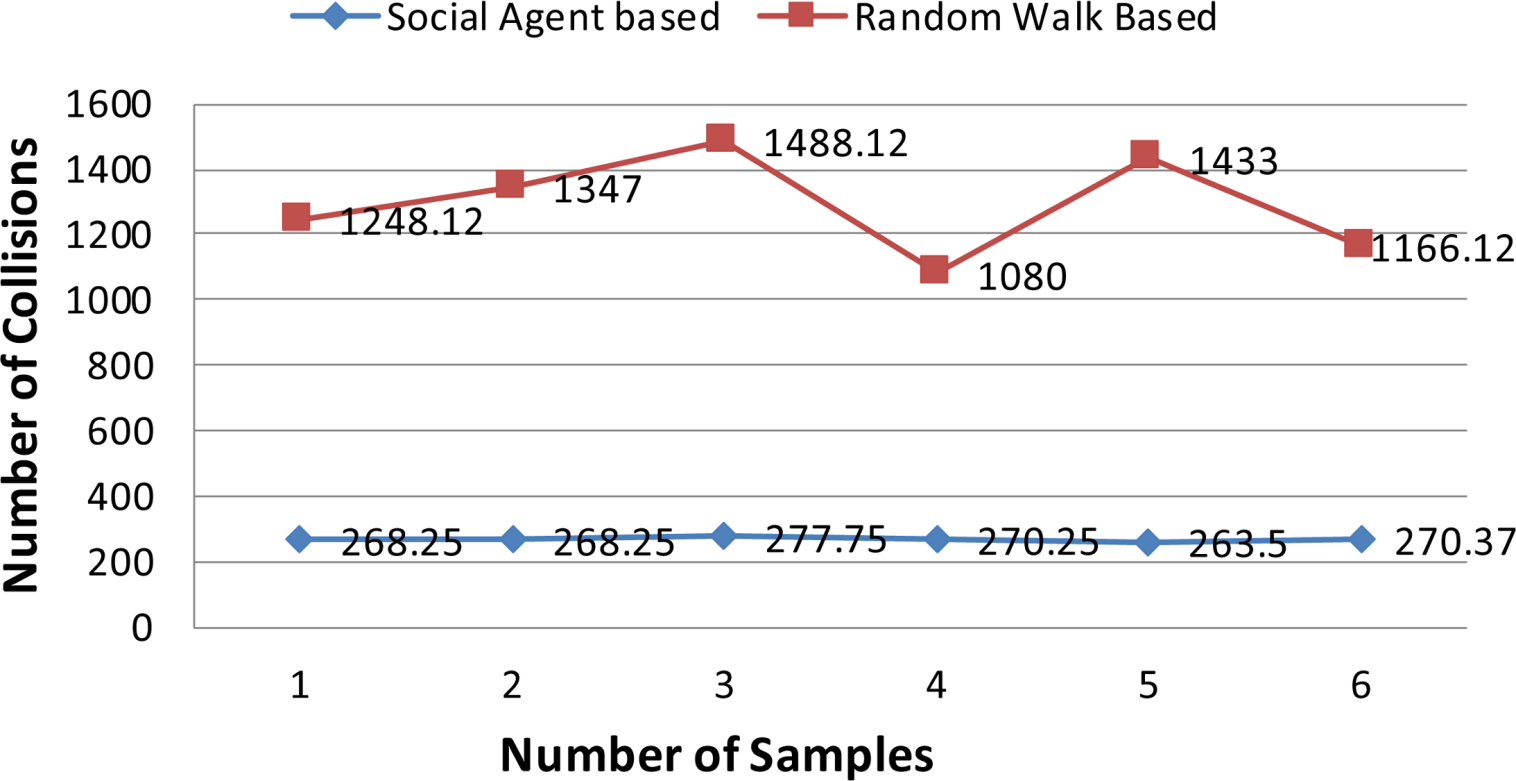
Graphical representation of test case 1.

**Fig 5:**
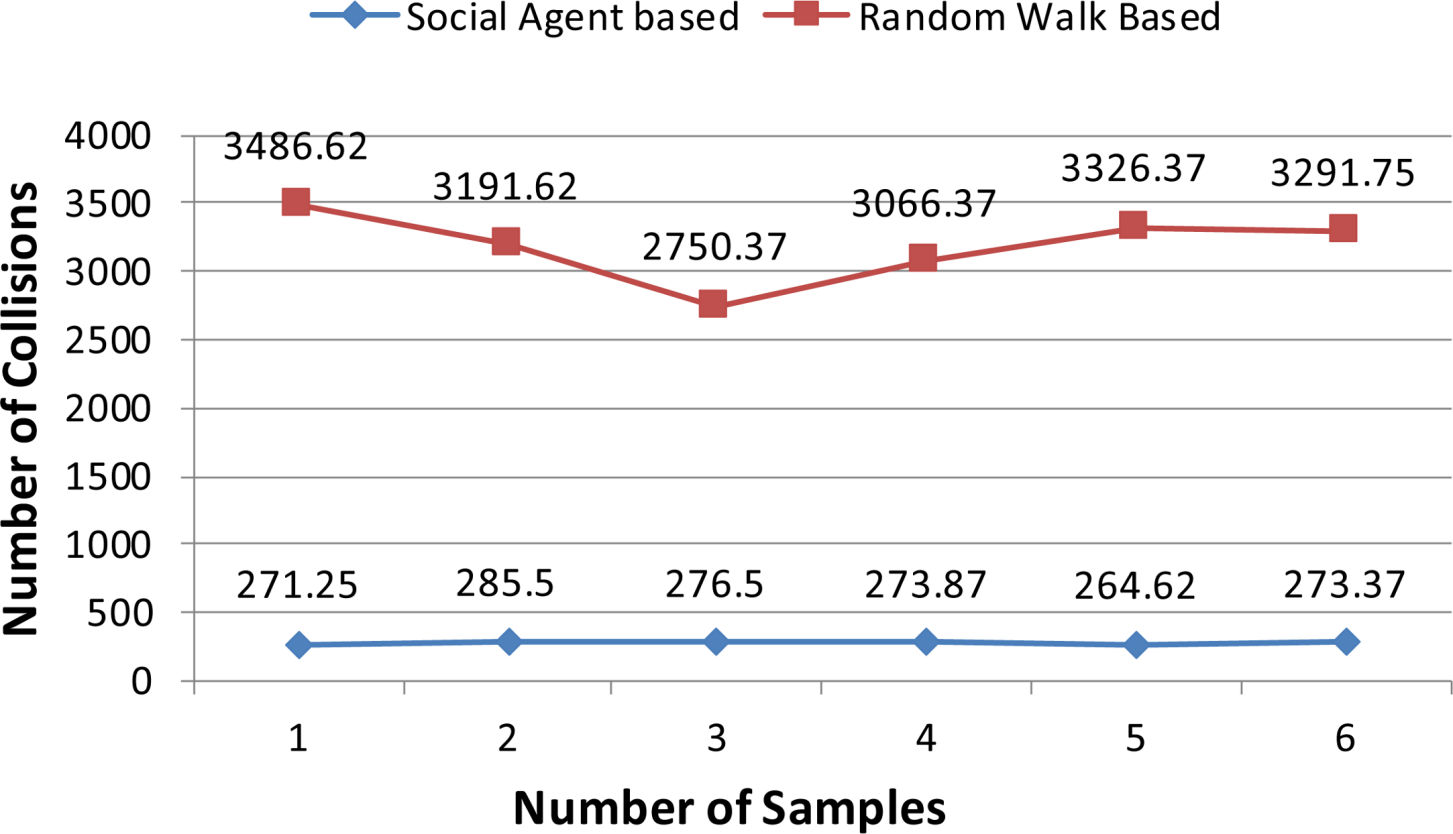
Graphical representation of test case 2.

**Fig 6:**
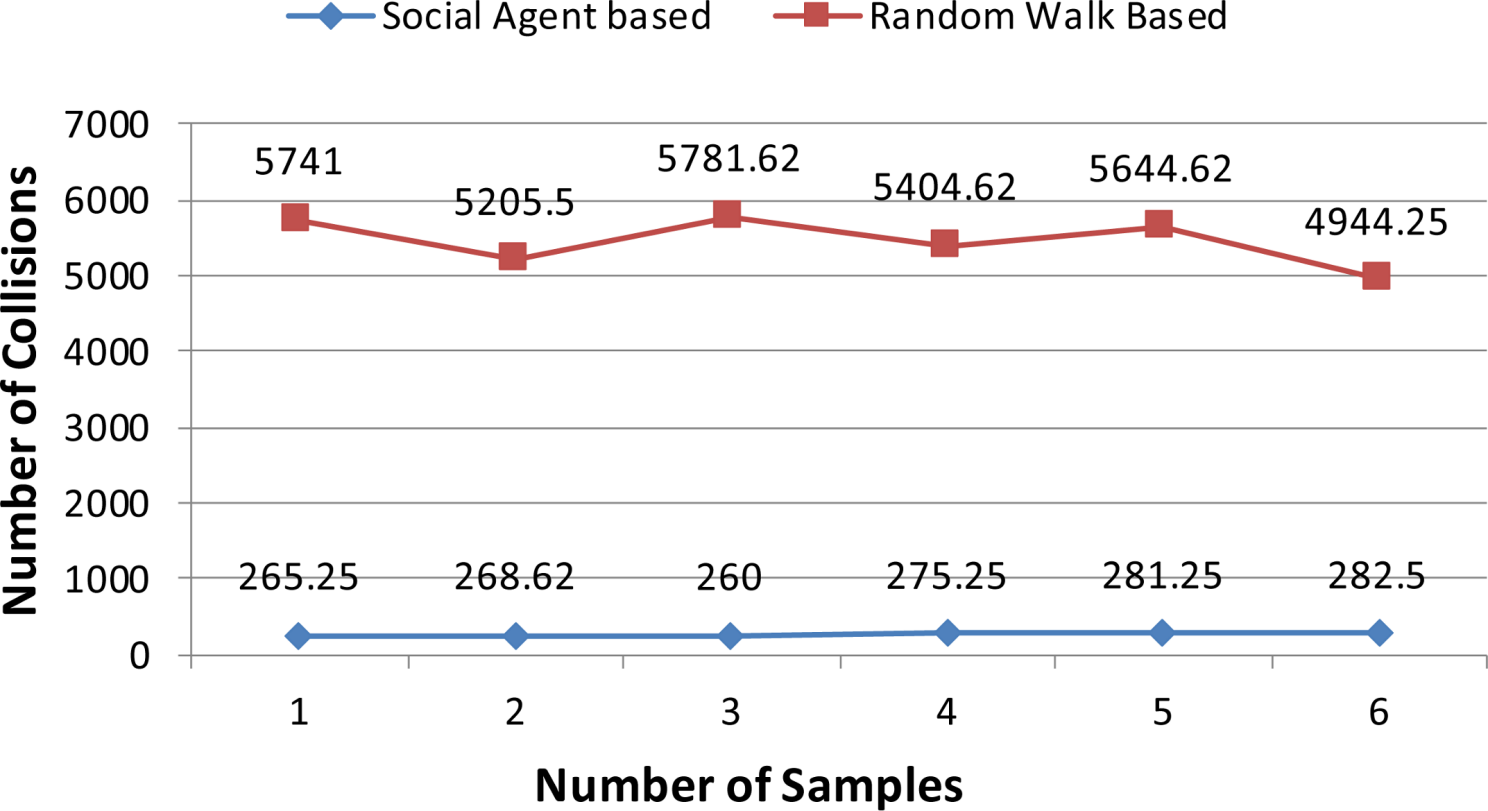
Graphical representation of test case 3.

**Fig 7:**
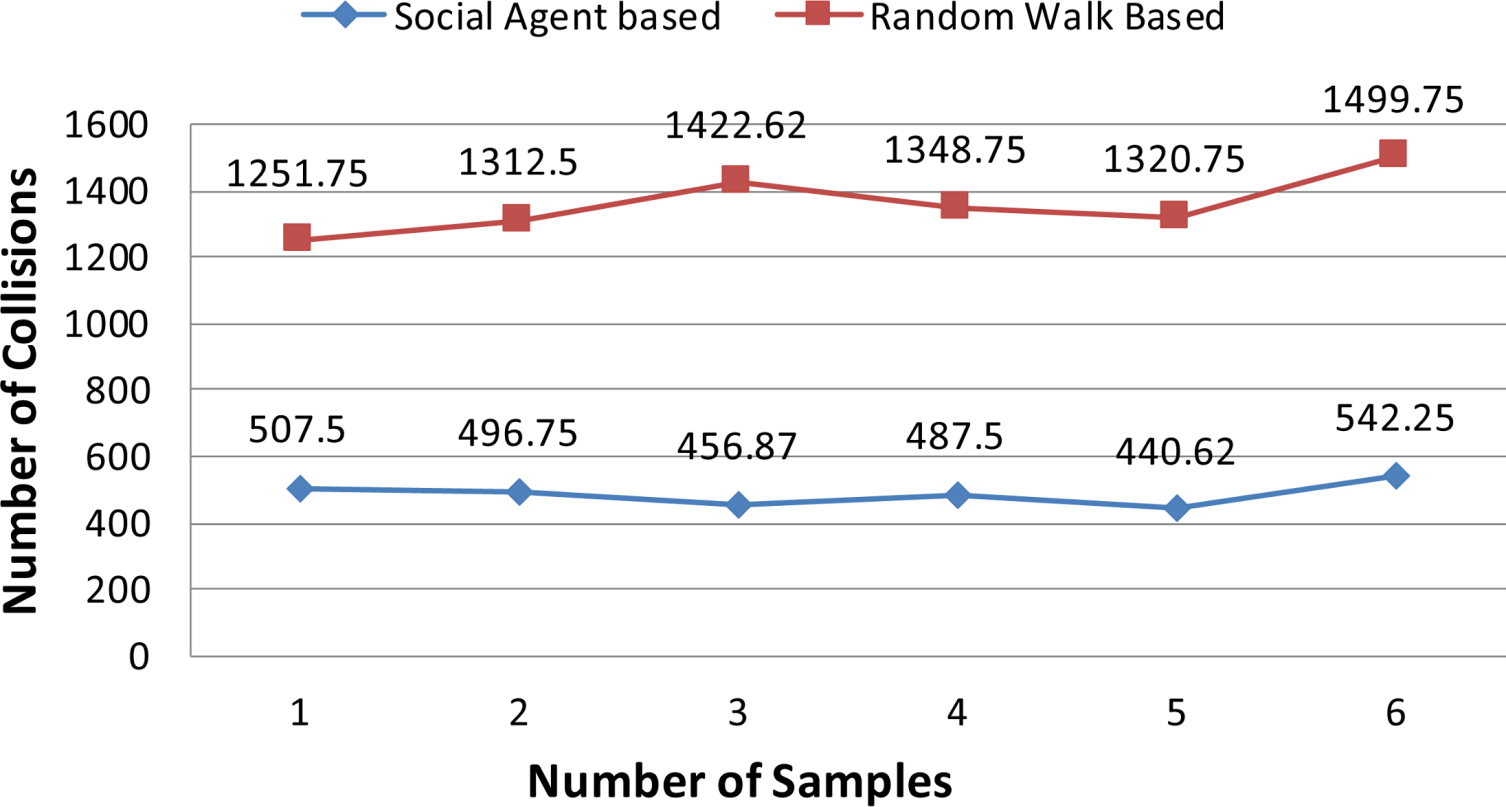
Graphical representation of test case 4.

**Fig 8:**
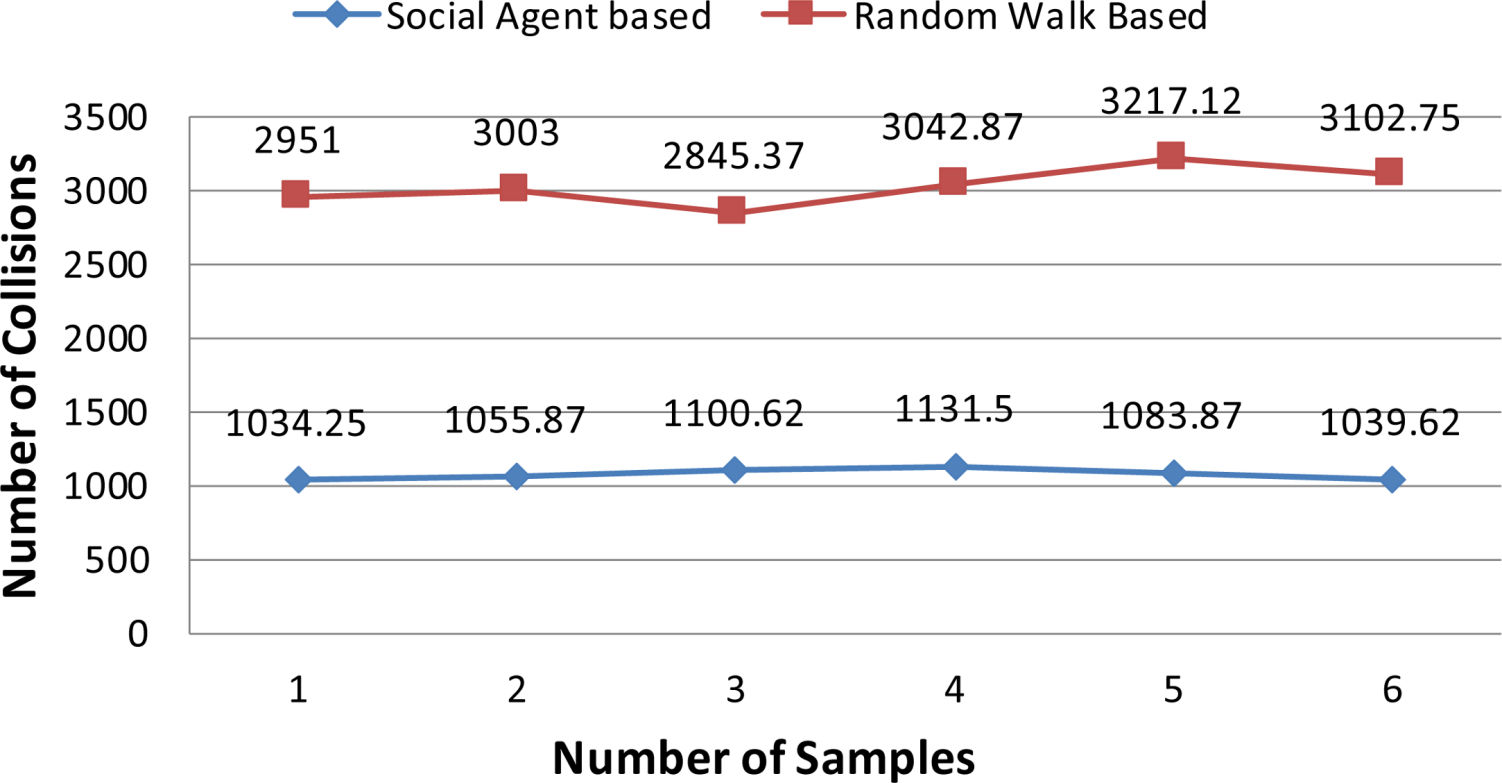
Graphical representation of test case 5.

**Fig 9:**
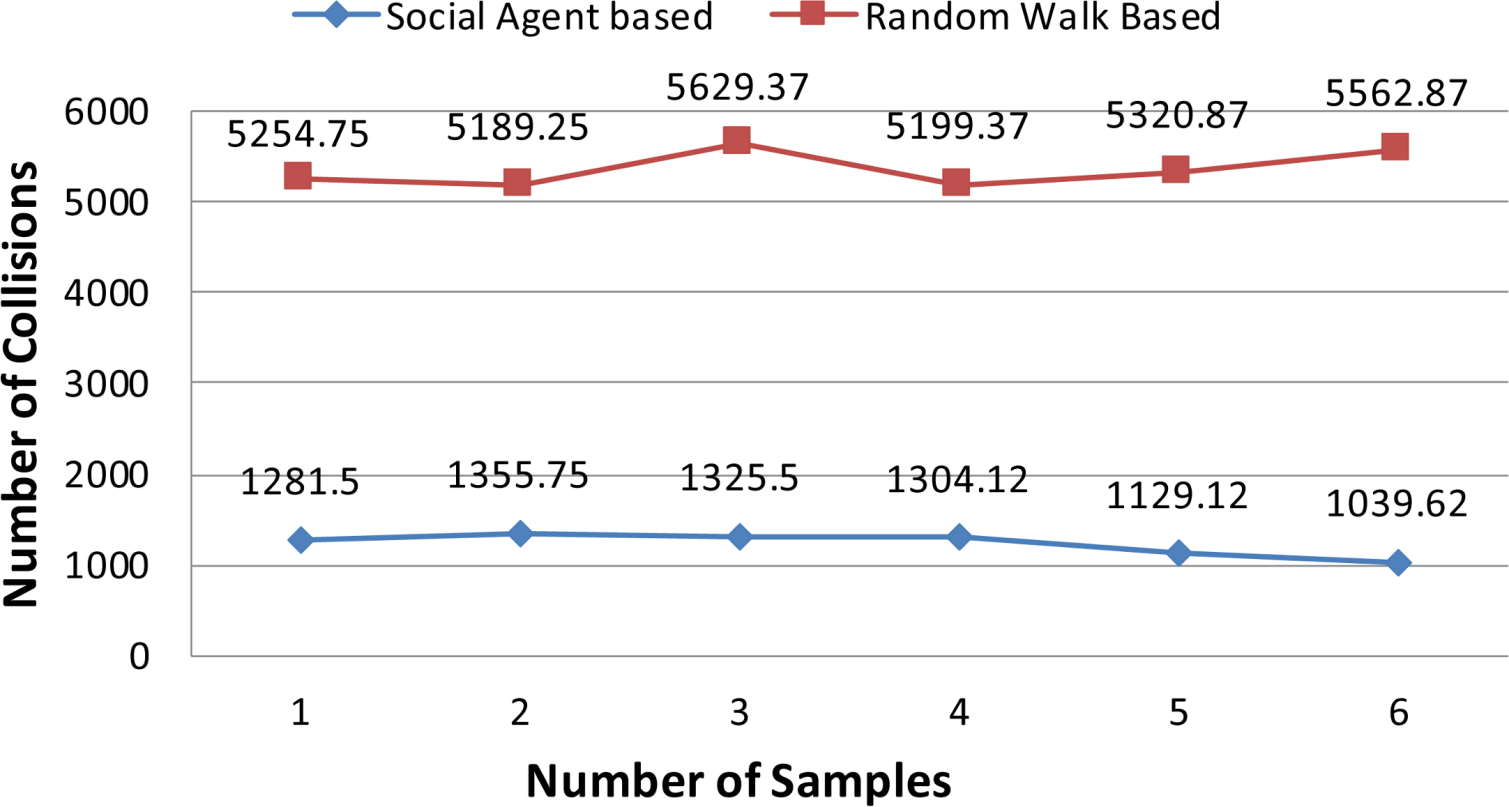
Graphical representation of test case 6.

In this section results of the simulation experiments along with detailed discussion are presented. Figs 4–6 present the results of the first three tests of an experiment set in terms of a mean number of collisions along their standard deviation values. From the Fig 4, it can be seen that the proposed social agent based collision avoidance scheme outperforms random walk based collision avoidance scheme when the total number of AVs is 40. In the first result, there are 1248 collisions, when the AVs follow the random walk pattern for travelling. However, using a social agent based technique the number of collisions decreased to the figure of 268.25. It means that the proposed technique helps AVs to avoid the collisions by having the know-how of each other's current position using Sensory and Artificial Thalamus module and mirroring module. In the same way, the 6^th^ entry of Fig 4 shows that using the proposed technique, there are only 270 collisions as compared to the 1166.12 collisions in the case of Random walk based movement of AVs. From the remaining Figs (5 and 6), it can be seen that the social agent enabled AVs outperform random walk based AVs in terms of fewer collisions. An interesting fact is that for random walk based AVs, the number of collisions increase with an increase in the number of AVs, whereas for proposed technique, the range of collisions does not cross the figure of 300 collisions. Figs 7–9 presents the simulation results of the last three tests of experiment set. If we recall last three tests of experiment set, Table 1, then these are different from the first 3 tests 1 in terms of minimum and maximum velocity range. Now the range has been set between 0.5 to 0.9. Whereas, the deceleration rate is same, i.e. 0.1 m/s^2^. In these simulation tests, the performance of social agent enabled AVs is compared with random walk based AVs in terms of collision avoidance. Fig 7 presents the comparison of both techniques for test case 4 having 40 red and 40 black AVs. In the first result, there are 1251.75 collisions when AVs use the Random walk pattern to travel. Whereas the number of collisions has been minimized using the social agent model and there are only 507.5 collisions. In the same way, the other values of Fig 7 are 1312.5, 1422.62, 1348.75, 1320.75, and 1499.75 for random walk based technique. Whereas for the Richardson’s arms race model technique these numbers of collisions are 496.75, 456.87, 487.5, 440.62, and 542.25 respectively. If we compare these results, then it can be seen that our proposed scheme have performed fewer collisions as compared to the Random walk based AVs. Further, the analysis of remaining Figs 8 and 9 proves that social agent based collision avoidance scheme outperforms Random walk based collision avoidance scheme. Now if we compare the results of the first three and last three tests of experimental set then it can be seen that Richardson’s arms race model with high velocity has a higher number of collisions as compared to the Richardson’s arms race model with low velocity. Hence, it can be observed very clearly that Richardson’s arms race model with low velocity and low deceleration rate can outperform the Richardson’s arms race model based collision avoidance technique with high-velocity and low deceleration rate. The dataset generated and used to draw these conclusions in the result of Random walk based and Social Agent Based collision avoidance simulations have been provided in the supporting files S1 Text, S2 Text, S3 Text, S4 Text,S5 Text, S6 Text, S7 Text, S8 Text, S9 Text and S10 Text respectively.

**Fig 10:**
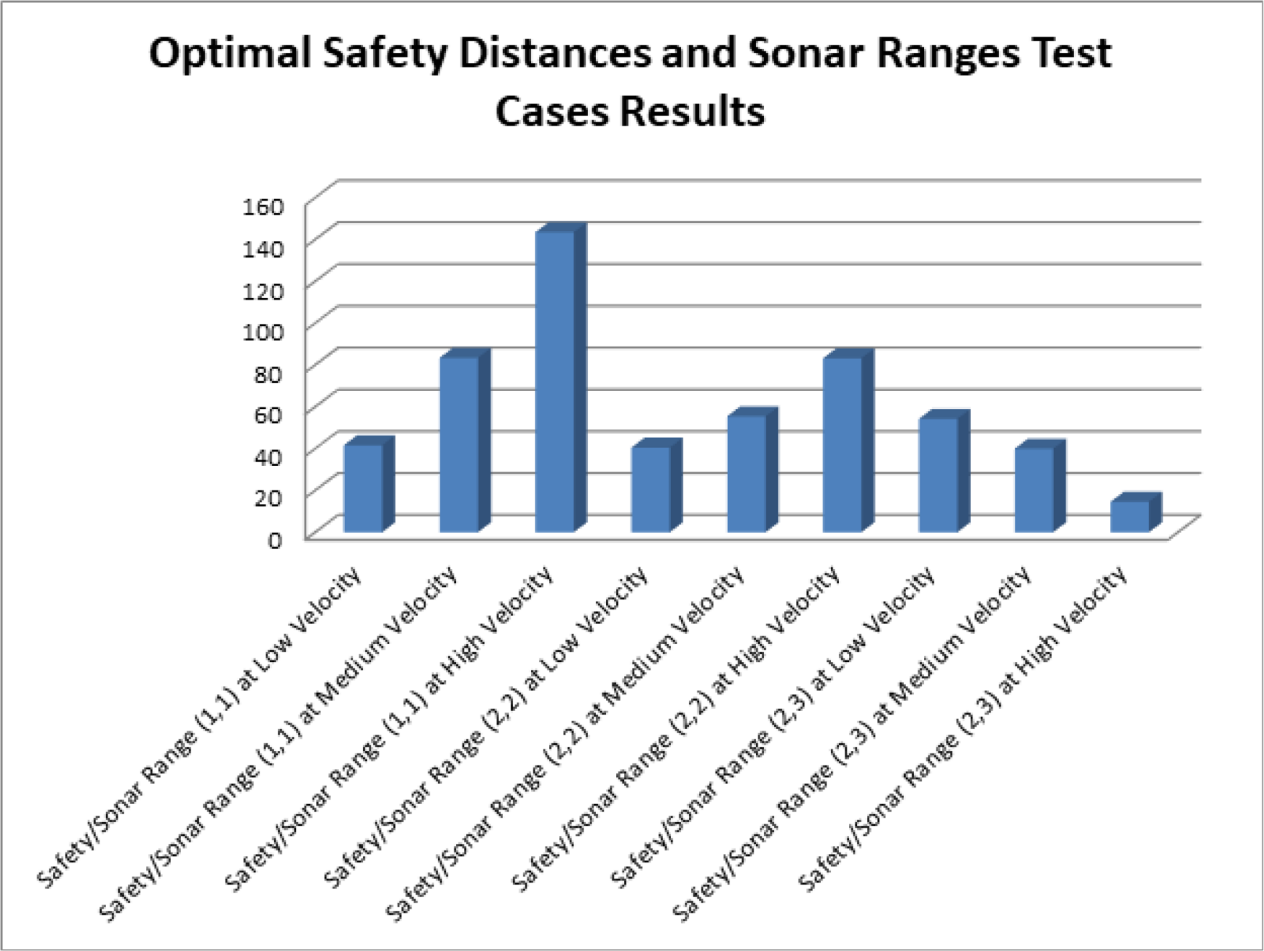
Infield experiment using flock like topology.

The results of the second type of experiments, which were performed to find out the optimal speed, safety distance and sonar range for least collisions in flocks like topologies have been presented in Fig 10. From the results it can be seen that when the safety distance and sonar range was set to 1 feet and AVs traveled at low velocity (0.1 m/s– 0.4 m/s) then the total number of collisions were in the range of 40. Whereas, using same safety distance and sonar range settings the number of collisions at medium (0.3 m/s– 0.5 m/s) and high speed (0.3 m/s-0.7 m/s) reached in the range of 80 and 140 respectively. In the next subset of experiments, the both safety distance and sonar range parameters were set to 2 feet. In a result, the number of collisions at low speed remained in the range of 40. However, the significant difference has been found for medium and high speeds. The number of collisions for medium speed decreased from the range of 80 to the 50 and for high speed, the number of collisions reached in the range of 80 as compared to the 140 previously. In the last three test cases, we tested another hypothesis that what will be the results, if we set the sonar range high as compared to the safety distance parameter. The results were very interesting for these test cases. However, this combination of safety distance and sonar range doesn’t work well when AV travels at low speed. However, at medium and high speed, the number of collisions reached in the range of 40 and 14 respectively. From these test cases, it has been concluded that it would be better to code the following optimal sonar values and safety distances in the autopilot of the AVs to have less number of collisions, while travelling in the flock like topologies. When the AV is traveling between 10m/s to 40m/s then it would be better to set its safety distance and sonar range to 1 feet each. If the AV, is travelling at medium (30 m/s 50 m/s) and high (50 m/s to 70 m/s) velocities then it would be better to set its sonar range greater than the safety distance. The datasets, generated in the result of these test cases upon which these conclusions have been drawn, are provided in the supporting files S11 Text, S12 Text, S13 Text, S14 Text, S15 Text, S16 Text, S17 Text and S18 Text respectively.

### Results and discussion of real time validation experiments

**Fig 11:**
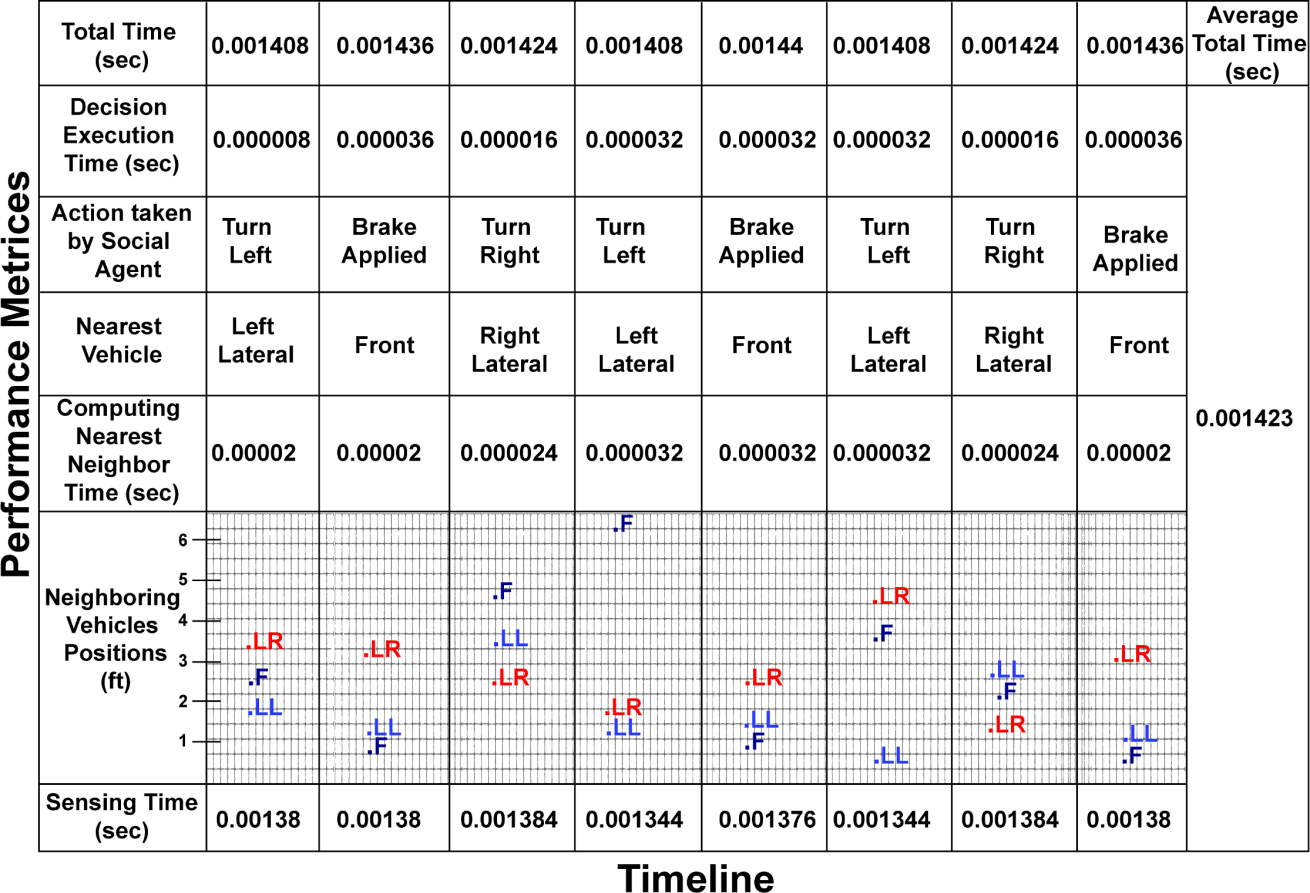
Results of in field experiments in terms of time taken by the social agent for the collision avoidance in the flock like topology.

The real time experiment is performed with three human-driven motorcycles and specially built AV installed with a social agent. Furthermore, Fig 11 4 presents the results of in-field experiments. Total 8 tests have been performed to validate the performance of the social agent. If we study the results of the first test then it can be seen that social AV takes 0.00138 seconds to sense the three neighbouring vehicles and found the front vehicle at the distance of 2.6 ft, and Lateral Left (LL) and Lateral Right (LR) vehicles in 1.8 and 3.2 ft respectively. In next step social agent takes 0.000002 seconds to compute the nearest vehicle and declared LL the nearest one. During the experiment, when the LL drifted towards AV and reached the preset safety threshold the mirroring module of social agent copied the drifting angle of LL and executed turn left manoeuvre in 0.000008 seconds. The total time taken by a social agent from sensing the neighbours to execute the collision avoidance manoeuvre is 0.001408 seconds. In the same way, the other tests prove the effectiveness of the proposed approach regarding collision avoidance in a very short time.

### Discussion and comparison with the state-of-the-art

As discusses in section 2, Motivation behind research work, we have not found any research work to addresses the presented problem. However, we have found a mirror inspired cooperative perception based collision avoidance scheme by Kim and Liu [32] which is close to our proposed research in a single aspect. Kim and Liu [32] utilised the concept of mirror neurons to propose the longitudinal and lateral motion control mechanism using cooperative perception. The presented model is a macroscopic model, which takes into account the overall behaviour of the AVs. Though the authors have claimed to use the human mirror Neurons to guess the intention of leading vehicles, it relies on cooperative perception. However, the intention aware mechanism regarding laterally moving vehicles has not been devised that help the AVs to optimise their latitude control and help them in avoiding lateral collisions. Furthermore, the cooperative perception has been utilised, which depends on the wireless medium. According to [32], cooperative perception is suitable in making short-term perspective driving the decision for hidden collision Avoidance but it doesn’t help in defining the longitudinal and lateral control mechanism, which helps the autonomous vehicles to avoid the collisions from the non-hidden neighbouring vehicles, travelling in side by side fashion. Furthermore, the cooperative perception between AVs has been supposed to be made using Wireless access for Vehicular Environment (WAVE) as a communication medium. According to [33], WAVE has not been found suitable to provide the reliable communication medium for increasing number of vehicles competing for the same channel within the same area. The real-time applications like road safety using cooperative perception require less than 200 milliseconds delay [34] but it has been noted by [35] that due to data contention in the control channel of WAVE, data packets have to be re-sent many times and as a result the safety message delivery time exceeds 1000 milliseconds. To measure the performance of IEEE 802.11n based Mirror Neuron Inspired Intention Awareness and Cooperative Perception Approach, we setup an experimental environment. The experimental platform consists of two toy AVs equipped with Arduino microcontrollers, GPS and wireless transceiver. To measure the performance of IEEE 802.11n based intention aware scheme, following metrics has been considered. ***Packet preparation time by sending Vehicle***, ***Average packet delay time between two vehicles*, *packet interpretation time by destination vehicle***, and ***Reaction time to avoid the collision***. The test results are presented in Table 4. From the first test result, it can be seen that the sending vehicle takes 0.600372 seconds to prepare the message packet and then forward it to the destination vehicle. The message packet reaches to the destination vehicle with the delay of 0.152 seconds. After receiving the packet the destination vehicle takes 0.402408 seconds to understand the message hidden in the packet. In the next step, the destination vehicle executes the collision avoidance manoeuvre in 0.000008 seconds. In this way, the total time taken by destination vehicle to avoid the collision is 1.154 seconds. We performed total eight experiments and it has been revealed that IEEE 802.11n based mirror neuron scheme takes 1.1109 seconds on average to avoid the collisions.

**Table 4:** Results of prototype experiments in terms of the time taken by the IEEE 802.11n based mirror neuron inspired intention awareness and cooperative perception approach [32] for the collision avoidance in the flock like topology.

**Total Time** **(sec)**	1.154788	1.17464	1.078712	1.037716	1.266761	1.019812	1.080626	1.074752	**Average** **Total Time** **(sec)**
**Action** **Taken** **Time (sec)**	0.000008	0.000036	0.000016	0.000032	0.000032	0.000032	0.000016	0.000036	1.110975875
**Packet** **Interpretation** **Time** **(sec)**	0.402408	0.40224	0.40034	0.402328	0.600365	0.402418	0.402245	0.40034	
**Packet** **Transmission** **Delay** **Time** **(sec)**	0.152	0.172	0.078	0.035	0.066	0.017	0.078	0.074	
**Packet** **Preparation** **Time** **(sec)**	0.600372	0.600364	0.600356	0.600356	0.600364	0.600362	0.600365	0.600376	

In contrast to this research, we presented the microscopic model of collision avoidance using mentalizing and mirroring neuron without relying on cooperative perception. In conclusion, the proposed social agent based AVs can avoid rear end and lateral collisions in a flock like topology in 0.001423 seconds as compared to wireless based intention awareness system which takes 1.154 seconds for the same purpose. Hence the proposed scheme can avoid rear end and lateral collisions, in a flock like topology, with the efficiency of 99.876% as compared to the IEEE 802.11n based existing state of the art [32] mirroring neuron based collision avoidance scheme. Last but not least, during this research, we have learned a lesson that the efficiency of the proposed system can be increased by exploring the mechatronic actuator controls like brake and steering and energy efficiency of the proposed system. In this aspect, the following state of the art proposed by Lv et al. [36] and [37] seems very useful. Lv et al. [37] have proposed a sliding mode control based efficient hydraulic brake system, which decreases the reaction time of hydraulic brake and if we use this research with our proposed system then its efficiency can be increased significantly. In another research work, Lv et al. [36] have evaluated the benefits of using the regenerative braking system, which ultimately helps in decreasing the energy consumption of autonomous vehicles. Hence the concept of regenerative braking system can be utilized along the sliding mode control based hydraulic brake system to enhance the reaction time and energy efficiency of the proposed system, which ultimately helps the proposed system to be more practical and efficient. Furthermore, a novel vehicle-traffic interaction method proposed by Sun et al. [38] can also be utilized for efficient energy management, while travelling in a flock like topologies during long distance trips.

## Conclusion

Artificial intelligence is the name of building machines, which act like human beings, by studying human beings. Autonomous vehicles are in town and no one can negate their importance. However, building collision free AVs is a challenging task. To address this, we proposed the concept of social AVs, which use the social interaction mechanism of human beings to avoid the potential collisions. Humans have special brain circuits that make them social and help them to interpret the intentions of other human beings and adapting the strategies to avoid the clashes. Inspired from this, we have proposed a concept of a social agent that helps the AVs to avoid the collisions. In addition, a mathematical model inspired by Richardson’s arms race model is proposed to emulate the social functions of the human brain like mentalizing and mirroring. The performance of the proposed social agent is compared, using extensive experiment tests, with Random walk based collision avoidance strategy and it has been found that the proposed social agent based collision avoidance strategy is 78.52% efficient than random walk based collision avoidance strategy and the practical validation results confirm that the proposed scheme can avoid rear end and lateral collisions with the efficiency of 99.876% as compared to the IEEE 802.11n based existing state-of-the-art research work. Furthermore, the simulation results have provided optimal parameters, like optimal sonar range and different optimal speeds suitable for avoiding the road collisions in different road traffic situations. This research might be suitable for AV vendors to reinvent the autopilot design. It will make AVs capable of coping with the current dilemma that how the AVs make themselves more trustworthy in terms of safe travelling.

## Supporting information

S1 Text (CSV)

S2 Text (CSV)

S3 Text (CSV)

S4 Text (CSV)

S5 Text (CSV)

S6 Text (CSV)

S7 Text (CSV)

S8 Text (CSV)

S9 Text (CSV)

S10 Text (CSV)

S11 Text (CSV)

S12 Text (CSV)

S13 Text (CSV)

S14 Text (CSV)

S15 Text (CSV)

S16 Text (CSV)

S17 Text (CSV)

S18 Text (CSV)
